# Physical Activity Supporting Connection to Nature, and Helping to Maintain Wellbeing during the Covid-19 Restrictions in England

**DOI:** 10.3390/ijerph18094585

**Published:** 2021-04-26

**Authors:** Liz O’Brien, Jack Forster

**Affiliations:** Forest Research, Social and Economic Research Group, Farnham GU10 4LH, UK; jack.forster@forestresearch.gov.uk

**Keywords:** physical activity, trees and woodlands, nature, wellbeing, nature connection, motivations

## Abstract

The Covid-19 pandemic and the restrictions put in place to prevent or reduce the spread of the disease led to anxiety, concern and stress for many people. In England restrictions varied at different times of the year, and throughout this time there was a lot of attention focused on the importance of exercise and on engaging with nature to maintain wellbeing. We undertook an online survey that ran for six weeks in June/July 2020 and gained a response from 2115 people, of which 25% were male and 74% female, with 35% aged 16–44 and 65% aged 45+. This survey focused on people who were already interested or engaged with nature. We explored whether being physically activity changed or not, if being active impacted people’s wellbeing and whether those who were active benefited from connecting to nature. We found that those meeting the government recommended levels of physical activity in the previous week, of 150 min, were more likely to maintain their overall wellbeing through feeling that the things they did in their life were worthwhile (*p* < 0.0001) and reported an increase in feelings of connection to nature (*p* < 0.0001). While those who did less than 30 min of physical activity in the previous week were less happy (*p* < 0.0001) and more anxious than usual (*p* < 0.0001). The research highlights the importance of physical activity and contact with nature and how these can play important roles in maintaining people’s everyday wellbeing under extremely difficult national circumstances.

## 1. Introduction

Covid-19 was identified in Wuhan in China in December 2019. It was declared a global pandemic by the World Health Organisation in mid-March 2020. By early April nearly 4 billion people worldwide were being told to stay at home by their respective governments to reduce the spread of the disease [1]. The unprecedented curtailment in people’s activities led to many people not being able to work, others worked from home, and many of people’s usual leisure activities associated with shopping, eating out, going to the cinema, theatre, gym, or going on holiday or mini-breaks were curtailed. With major concerns about the disease and fewer options of activities that people could undertake, coupled with strong encouragement for people to get outdoors to exercise—a focus on connecting with nature became more prominent on social media. The World Economic Forum’s Covid action platform [2] argues that focusing on nature can contribute to an understanding of where pandemics come from but can also highlighted how nature can have an impact on people’s wellbeing. With over half of the global population living in urban areas, access to urban and peri-urban greenspaces became increasingly important [3].

A study of green space use during the crisis in Oslo (Norway) found an estimated 291% increase in outdoor recreational activity compared to a 3-year average [4]. The study used mobile tracking data to explore this change and highlighted increased use of city parks, peri-urban forests as well as protected areas; emphasising the importance of green space near to where people live. An international exploratory study focused on six European countries, and the use and perceptions of urban green space and the effects of Covid-19, found increases in walking to nearby green spaces or along tree lined streets along with people missing the social connections of meeting others outdoors, and generally spending more time outdoors. The study found urban green spaces provided people with solace and respite as well as places to relax and exercise [3]. In another study of a large peri-urban forest near Bonn in Germany expert interviews with forest managers highlighted both an increase in visitor numbers, visits being made at different times of day than previously, and new types of visitors accessing the forest [5]. A further study in Vermont (USA) found increased participation in walking, wildlife watching and relaxing outside alone [6]. In England a large scale “People and Nature Survey” in April 2020 found that nearly 50% of adults had spent time outside in green and natural spaces in the previous 2 weeks, with urban green spaces being the most visited type of nature, and the majority of adults (59%) with a garden stating that having a garden was very important to them. This survey (which is longitudinal) and its initial results took place while England was in lockdown and when people were only allowed out to exercise for one hour a day from the end of March until mid-May [7]. By October 2020 when fewer restrictions were in place 62% of adults in England had visited a green or natural space in the previous 2 weeks with 29% reporting staying at home to reduce the spread of coronavirus [8]. Shoari et al. [9] quantified access to public gardens and parks in urban England and Wales, they found that 25.4 million people live within 1000 m of public parks and gardens in urban areas of England and Wales. They also suggested that if people in urban areas all visited their nearest parks at the same time, in 50% of those parks people would not be able to maintain the advised social distance (4 m^2^) due to the coronavirus pandemic. A survey in Japan found those on higher incomes more easily accessed greenspaces than those on lower incomes who faced difficulties with transportation and time constraints [10].

A wide range of existing evidence illustrates that human relationships with nature provide many services and benefits not only provisioning, regulating and supporting identified through an ecosystem services framework but also many cultural services that can lead to a broad range of wellbeing benefits [11,12]. Due to the benefits identified in many studies, the overall aim of this research was to explore changes in engagement with nature brought about by Covid-19 with a particular focus on physical activity. The focus of the research was on people with an existing interest in, and engagement with, nature in order to explore whether this interest and engagement changed or not during the Coronavirus restrictions. We chose this type of sample to explore whether this existing connection could help support people during the Covid-19 pandemic. The results can also be explored and potentially compared with surveys of representative samples of the population to identify differences and similarities. We would expect that a sample of people already engaged with nature might find a greater level of support from engaging and connecting with nature during difficult times than the general population. Ward Thompson et al. [13] found that those who visited nature during childhood were more likely to visit as an adult and more frequently than those who did not visit as a child. Pritchard et al. [14] in a meta-analysis of nature connectedness and eudaimonic wellbeing found a small but significant relationship between the two, with those being more connected to nature having greater eudaimonic wellbeing. Other studies highlight how nature can be viewed as an escape from the pressures and concerns of everyday life [15,16] and during Covid-19 this became especially important for many. We focused on physical activity in particular as it can help to protect against anxiety [17], lower the risk of experiencing stress [18] and has some positive associations with happiness [19,20].

Specifically, we explored the following research questions: (1) Were there any changes in people’s physical activity due to Covid-19 and was this linked to wellbeing? (2) Were they any changes in people’s engagement and feelings of connection with nature due to Covid-19? (3) What barriers did people report that prevented them engaging with nature during Covid-19?

## 2. Materials and Methods

An online survey was developed to identify whether there were changes in people’s physical activity levels and whether this was linked to wellbeing, connections with nature and barriers to accessing nature. The survey focused on people in England with an existing interest in nature and in woodlands in particular. In order to reach this engaged group, the survey was publicized by Forestry England, which is responsible for managing and promoting use of the nation’s forests in England, through its newsletters in June and numerous times through its Facebook, Instagram and Twitter social media presence. The survey was the most clicked on item in the Forestry England June newsletter (pers comm). An incentive of seven prizes were offered and all those who completed the survey, were entered into the prize draw in which they could win one of three memberships to Forestry England sites or one of four Go-outdoor clothing vouchers. As only seven respondents would gain a prize from completing the survey, we did not consider that this would have acted as a large incentive to survey completion or impacted on the type of respondents likely to get involved in the survey. In order to be entered into the prize draw respondents had to leave their email address, we outlined that this would only be kept until the prizes were drawn. Respondents were also asked if they would be willing to be interviewed and if so to leave a contact email or phone number, and their post code to explore whether they lived in affluent or deprived parts of England. We outlined that their data would be confidential, none of the data would be shared with a third party, respondents would remain anonymous to each other and the wider public, and that the information would be stored and analysed in line with the Data Protection Act 2018. Sensitive data was stored securely in line with the ethical guidelines of Forest Research [21].

The survey ran (using Smart Survey as the survey platform) from mid-June until the end of July 2020. In terms of Covid-19 restrictions in June people were allowed to meet up to six people outdoors and in early July non-essential shops opened along with pubs.

The main focus of the survey was to explore any changes positive or negative in the following (see Supplementary Materials for the survey questionnaire):

- Engagement with and visits to nature
- Motivations for visiting nature
- Wellbeing benefits gained from nature
- Feelings of connection to nature
- Barriers to accessing nature
- Physical activity in the last seven days
- Overall personal wellbeing

The survey included mainly closed questions but also included one open question and four opportunities to provide a qualitative comment; these, over 3000 comments along with 25 interviews will be published in a separate paper. We asked specific questions related to the above categories and asked respondents if these had changed or increased due to Coronavirus on a five-point Likert scale. The Office for National Statistics (ONS) 4 personal wellbeing questions were also used. These are part of the wider measuring of national wellbeing in the United Kingdom (UK) [22]. The four questions cover life satisfaction, feelings of life being worthwhile, as well as anxiety and happiness. We added two further questions to explore whether people felt their happiness or anxiety had changed since the Covid-19 restrictions came into place.

In total 2115 responses were completed and submitted (Table 1). There were 1726 partially completed surveys that were started by respondents but not submitted and therefore we have not included these in our study. The majority of age groups were well represented with more than 10% of respondents in each category, apart from the 16–24-year age group (*n* = 19). Therefore the 16–24-year age group was combined with the 25–34 age group to provide a suitable sample size for analysis. Three quarters of respondents were female, and the majority were white. Just over 60% were from urban postcodes, 20% peri-urban, and 20% from rural postcodes. 12% of respondents lived alone, approximately 50% lived in two person households and nearly 60% lived in households with no children under-16 years of age. Those living in the least deprived areas in England were over-represented in the sample, this was identified by people’s postcode. The Index of Multiple Deprivation covers seven domains from income to health and is applied to over 32,000 small areas in England with the most deprived areas being scored at 1 and the least deprived scored at 10 [23].

This survey did not aim to capture a representative sample of the population in England, it aimed to capture people who had an existing interest in nature and particularly trees and woodlands. There was an over representation of women in the sample which is not dissimilar to the sample that engages with Forestry England, particularly those following Forestry England on Facebook. Forestry England’s July 2020 Facebook followers were 73% women, with the largest group being aged 35–44 (43%); its Instagram followers in June were 56% women and its Twitter followers in the same month were 57% men [24]. The sample involved in this study was primarily female and white. The findings provide insights into physical activity, connection to nature and wellbeing for those connected to nature already. This limits the generalizability of the study to the population as a whole but does provide evidence for the population of people interested in nature.

### Data Analysis

All analysis was conducted in R [25], with data cleaning and manipulation conducted using R package “dplyr” [26]. Postcode data were cleaned and matched to Index of Multiple Deprivation and urban rural classification data via the Office for National Statistics publicly available databases. Urban rural classifications for England/Wales were aligned as follows: England/Wales “Urban city and town” = “Urban”, “Urban major conurbation” = “Urban”, “Urban minor conurbation” = “Urban”, “Urban city and town in a sparse setting” = “Peri-urban”, “Rural town and fringe” = “Peri-urban”, “Rural town and fringe in a sparse setting” = “Rural”, “Rural village” = “Rural”, “Rural village in a sparse setting” = “Rural”, “Rural hamlets and isolated dwellings” = “Rural”, “Rural hamlets and isolated dwellings in a sparse setting” = “Rural”; Scotland 6-fold classification: “1” = “Urban”, “2” = “Urban”, “3” = “Peri-urban”, “4” = “Rural”, “5” = “Rural”, “6” = “Rural”).

Statistical methodologies were as follows: Each main question was analysed separately, with sub-questions treated as factors within the main question analysis. Although many questions had a binary response (e.g., yes/no), some were on an ordered scale (e.g., increase, same, decrease); lack of convergence for cumulative linked mixed effects models resulted in ordinal responses being aggregated into binary categories (e.g., increase vs. same/decrease) and analysed similarly to all other binary responses. For each question, a generalised linear mixed effects model [27] was applied to the data, with the main effects of sub-question and key demographics (physical activity levels, change in physical activity post Covid, gender, age, household size (under/over 16s), index of multiple deprivation and urban/peri-urban/rural location) as predictors. The model also included a two-way interaction between each sub-question and each demographic, and a three-way interaction between sub-question, age and gender. Gender data had to be restricted to male/female due to small sample sizes in other groups. The 16–24 age group had to be combined into a 16–34 age group due to small sample sizes. Individual user ID was included as a random effect in each model, to account for respondent-specific effects across the multiple sub-questions.

For wellbeing questions, where there were no sub-questions, the ordered 0–10 scales were analysed using ordinal logistic regression models [28] with the same key demographics as above. For happiness and anxiety change, the scales (−10 to 10) were grouped to form an 11-point scale (e.g., −10/−9 grouped together) to aid model convergence.

Statistical significance of main effects and interactions were determined using analysis of deviance (Wald Chi square tests for GLMMs, likelihood ratio Chi square tests for ordinal logistic models, Fox & Weisberg, 2011) [29] and non-significant interactions and main effects dropped from the final models. Here and throughout, a conservative *p* value of 0.01 was used as the significance threshold. Post hoc estimated marginal means and contrasts were calculated for significant effects (Tukey’s Honest Significant Difference multiple comparison adjustments, [30]). For each question key results were displayed graphically, with all global models’ significance tests and post hoc tests stored in data tables for future reference.

## 3. Results

### 3.1. Physical Activity Level Changes

The majority (59%, *n* = 1153) of people who were active (150 min in the previous 7 days) during lockdown stated that this was “much more” or “a bit more” than they usually did, with 24% stating that they were much more active. However, those who did less than 30 min of physical exercise in the previous 7 days were more likely to state this was “a lot less” or “a bit less” than usual (52%, *n* = 183). There was a significant interaction between changes in visiting green and natural spaces before and during lockdown and physical activity (Wald chi-square = 77.3, df = 30, *p* < 0.0001): less active respondents were more likely to reduce their visits to popular types of nature including: (1) fields, farmland, countryside (−20%), (2) rivers, lakes, canals, (−18%), (3) urban greenspace (−16%), (4) woodland or forest (−24%; percentages represent difference in decline between the least and most active groups).

**Figure 1:**
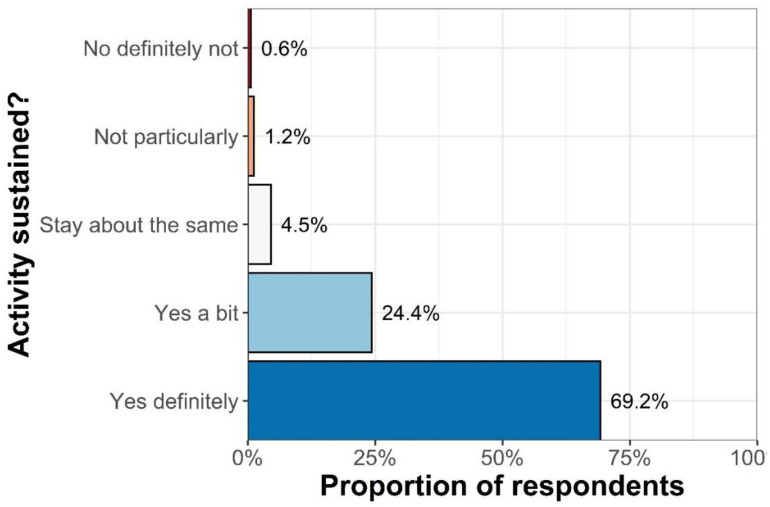
Likelihood of sustaining changes in individuals who had increased their outdoor physical activity during Covid restrictions (*n* = 1232).

Over two thirds (69%, *n* = 1232) stated that they would definitely sustain the increases in outdoor physical activity changes they had made in the long term (Figure 1). There were differences in these results by changes in physical activity (both indoor/outdoor), with those who had increased their physical activity significantly more likely to state that they would sustain changes (Wald chi-square = 30.0, df = 4, *p* < 0.0001).

### 3.2. Wellbeing and Physical Activity

Using the Office for National Statistics four personal wellbeing questions and the two extra questions on changes in anxiety or happiness levels due to Covid-19, we found that there was no significant change in happiness for those who were more physically active during lockdown (median score during restrictions = 8; IQR (inter-quartile range) 7–9), but a decline in happiness for those who were less active (median score during restrictions = 6; IQR 4.75–7.75, LR chi-square = 138, df = 4, *p* < 0.0001). The same outcome applied to anxiety with those doing much less activity than they usually did having a small but significant increase in anxiety (median score during restrictions = 5; IQR 2–7) versus more active groups (median score during restrictions = 3; IQR 1–6, LR chi-square = 28.9, df = 4, *p* < 0.0001).

In terms of how people feel about how worthwhile the things they do in their life are, those doing much less physical activity than usual during lockdown reported lower worthwhile scores than those doing the same or more activity (LR chi-square = 44.8, df = 4, *p* < 0.0001). Those who were more physically active had higher worthwhile scores (LR chi-square = 50.5, df = 3, *p* < 0.0001).

### 3.3. Changes in Engagement and Feelings of Connection to Nature for the Physically Active

Overall, more than two thirds (*n* = 2107) of people reported an increase in “time taken to appreciate nature”, “level of happiness when in nature” and “feelings of connection to nature” during the Covid restrictions. Women were significantly more likely to report an increase in nature connection than men (Wald chi-square = 17.3, df = 1, *p* < 0.0001, estimated marginal means of 86% and 75% for women and men respectively). There was also an increase in people’s appreciation of trees in different settings i.e., in woodlands, along footpaths/waterways, in the street, in people’s garden and in local parks. Again women were significantly more likely to show an increase in appreciation of trees and woods than men (Wald chi-square = 12.3, df = 1, *p* = 0.0005, estimated marginal means of 63% and 48% for women and men respectively, averaged across all sub-questions). There were differences by location and sub-question here (Wald chi-square = 25.3, df = 8, *p* = 0.0014), with a greater increase in the appreciation of trees in local parks for those in an urban versus rural settings (27.3% difference; *p* < 0.0001).

Those who had increased their physical activity during Covid-19 were significantly more likely to report an increase in feelings of connection to nature (Wald chi-square = 118, df = 4, *p* < 0.0001), and were more motivated to visit nature for a suite of reasons, not only to be active but also to explore and connect with nature as well as learn something new or challenge themselves (Wald chi-square = 253, df = 4, *p* < 0.0001). Those who had increased their physical activity during the Covid restrictions visited woodlands significantly more than those who were less active (Wald chi-square = 96.2, df = 4, *p* < 0.0001, approximately 1–2 additional visits per week for more active versus no additional visits for less active individuals) and were significantly more likely to report increases across a range of activities, including being in their garden, viewing nature/wildlife and visiting other nature areas (Wald chi-square = 310, df = 4, *p* < 0.0001). Those who had increased their physical activity were also more likely to report benefits from engaging with nature (Wald chi-square = 260, df = 4, *p* < 0.0001).

### 3.4. Barriers to Engaging with Nature during Covid-19

**Figure 2:**
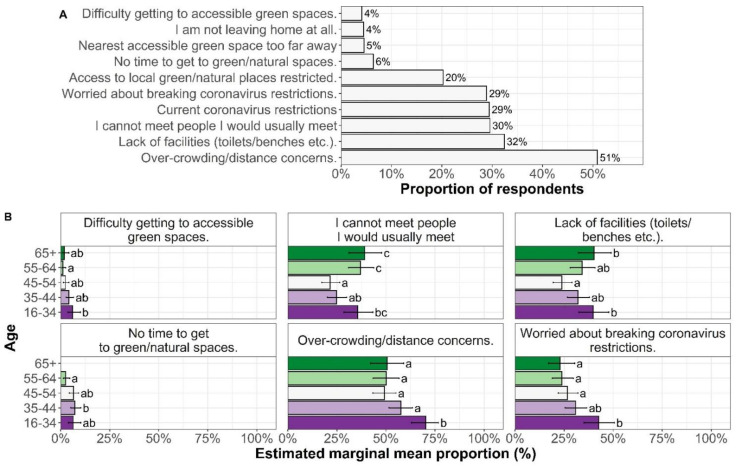
(**A**) Barriers to engaging with nature following the Covid-19 restrictions (*n* = 2115); (**B**) Estimated marginal means for barriers by age group. Error bars = 95% confidence intervals; lettering indicates significant differences, within a panel, where categories do not share a letter, they are significantly different at a *p* < 0.05 level (with Tukey’s HSD adjustments for multiple comparisons). Only barriers with significant differences by age group are shown.

The key barrier to engaging with Nature during the Covid-19 restrictions was concerns about overcrowding and not being able to keep a distance from others, which was reported by more than 50% of respondents (Figure 2). Households with under 16-year-old children were significantly (*p* = 0.0112) more likely to state that “not being able to use the facilities they needed” was a barrier than those households with no children present. A third of people reported “not being able to use the facilities they needed”, “not meeting people they usually would”, “current restrictions”, and “being worried about breaking restrictions” as barriers to engaging with nature. Individuals doing less physical activity during the restrictions were more likely to report across all barriers. However, there was no interaction between this and specific barriers; there is therefore no evidence of specific reasons for individuals reducing their physical activity during lockdown (e.g., if reduction in activity were due to shielding, we would expect a noticeable increase in individuals reporting “I am not leaving home at all”). There was a strong significant interaction between specific barrier and age (Wald chi-square = 104, df = 36, *p* < 0.0001), with post hoc testing by sub-question (shown in Figure 2) indicating that this was partly driven by younger people (16–34) being more likely to report not being able to keep their distance and breaking restrictions than older age groups.

## 4. Discussion

This research found that those who were physically activity in the previous week were more likely to sustain their overall wellbeing and feel more connected to nature during the Covid-19 pandemic. This work contributes to the growing body of evidence focused on the impacts of physical activity and issues of connection to nature during the various Covid-19 restrictions.

### 4.1. Physical Activity

In this study we found that those who were physically active to the recommended level of 150 min per week in the past 7 days were more likely to state that this was much more or a bit more than they usually did. There might be a number of reasons related to Covid-19 that explain this. Some respondents may have had more time due to not being able to work (i.e., they were furloughed—granted temporary leave of absence from work), or they saved time by working at home and therefore were not commuting. The average commuting time to work by train in Britain was 59 min in 2017 [31]. There was a strong focus in England in the first lockdown, that ran from late March until mid-May when people were only allowed out for 1 h a day to exercise, on making the most of that time and the importance of being active. This may have encouraged some people to be active and got them into the habit of being active when there was often little else to do as part of people’s leisure time. The Chief Medical Officer for England stressed the importance of exercise for health outdoors for those allowed out but also advocated indoor exercise for those who were shielding (i.e., those most at risk from coronavirus) and were advised not to go outside [32].

For those who did less than 30 min of physical activity in the last 7 days and for whom this was much less or a bit less than usual, they may have also been facing a range of issues. For example, over 2 million people were shielding in place in England until the end of July 2020 because they were considered at high risk [33]; which meant they were advised to stay indoors. However, this does not appear to be the case in this study. Others may have been working longer hours due to working in sectors that became busier including health, social care and food retail. While others may not have been able to get to local suitable spaces for exercise or felt that any spaces they could visit would be too busy and crowded. Parents and carers with children also had to spend considerable amounts of time on home schooling their children.

The Active Lives Survey run by Sport England found that from mid-March to mid-May those undertaking 150 min of physical activity per week dropped by 7.1% to 58.2% and those who were inactive (doing less than 30 min per week) rose by 7.4% to 30.4%. In this period walking for leisure was the primary activity people undertook followed by fitness activities, as many in the fitness sector switched to digital and self-led fitness classes [34]. A systematic review of changes in physical activity and sedentary behaviours during the Covid restrictions found physical activity decreases were reported in sixty-four of the sixty-six studies in the review, and sedentary behaviours increased [35].

### 4.2. Connecting to Nature

One positive aspect that has come out of the Covid-19 crisis is that more notice has been taken of the importance of nature for people’s wellbeing. Surveys, other than this one, highlight similar results to this research in terms of physical activity, wellbeing and connection to nature. For example, a survey undertaken on behalf of the Royal Society for the Protection of Birds (RSPB) [36] in England in May 2020 found strong support across age and income for the importance of nature for health and wellbeing, and particularly for nearby nature close to where people live. The survey also highlighted potential inequalities between households with high and low incomes in terms of access to nature. The People and Nature Survey in England (sample of 25,000 people every year and run on a continuous basis) results in November 2020 [8] show that 43% of adults in England were spending more time outside than before Covid-19, 27% were noticing nature and wildlife more, while 31% were exercising more outdoors than before Coivd-19. A survey for the Ramblers organization which focuses on walking also had a survey carried out on its behalf of over 2000 adults across Britain. This survey found that people felt the places where they lived were not green enough, and they felt green spaces were good for wellbeing and physical health, and people intended to be more active in the future [37]. Lockdown in England in late March to mid-May also coincided with the spring and a spell of bright and warm weather. The Met Office, which is the national meteorological service, stated that April 2020 was the sunniest on record for the United Kingdom (UK) as a whole, and the UK had one of its hottest summers on record [38].

### 4.3. Wellbeing, Physical Activity and Connection to Nature

There was a small but significant increase in anxiety and the same in terms of a decrease in happiness for those who were doing less physical activity and were now inactive (i.e., doing less than 30 min per week). A number of studies have highlighted the relationship between physical and mental wellbeing. For example, Harmer et al. [18] found daily physical activity was associated with lower risk of psychological distress and this included different types of physical activity from sports to housework and gardening. Petzold et al. [39] suggests that physical activity might have a preventative effect on people developing anxiety disorders. While, McDowell et al. [40] in a study of Irish adults found that meeting the physical activity guidelines of 150 min or more of physical activity a week resulted in 13.5% lower odds of anxiety. Other studies have found similar results that engaging in physical activity can protect against anxiety: from research in South Korea [17], in a systematic review [41] and in a World Health Study survey [42].

Those doing the same or more physical activity showed no significant change in happiness or anxiety levels. Studies have found a positive relationship between physical activity and happiness, with a systematic review suggesting that mediators for this relationship might include social functioning and health [43]. A study using the Eurobarameter data from 15 countries found that when compared to inactive people there was a positive association between physical activity and happiness, with walking and vigorous activity having small positive associations [19]. Overall, however these studies call for further research to explore causation and mediating factors.

This study showed that those who were meeting the recommended levels of physical activity were more likely to feel connected to nature, visit woodlands more, and gain benefits from engaging with nature. This may be due to these respondents spending more time in nature due to potentially having some more time because of changes in working practices. It may also be linked to the increased appreciation for nature noted in a number of recent surveys in England and in the wider UK, as well as people seeking some solace in engaging with nature at a time when they were anxious and concerned about Covid-19 and its impacts on society, and their friends and family [44]. Those who were more active also felt that their lives were more worthwhile, and evidence suggests that being active can foster feelings of confidence, self-worth and provide people with a feeling of achievement [20,45].

### 4.4. Implications for Policy and Practice

This study adds to the growing body of evidence concerning the importance of physical activity for people’s wellbeing and how being active in nature can be important in helping people feel connected to nature and gain multiple benefits from that connection. There is some research that highlights the added benefits of people undertaking physical activity outdoors in terms of feelings of escape and freedom, feeling close to nature and sensory stimulation [12,20].

A very good response rate to the survey was achieved with the support of Forestry England which publicized the survey through its newsletter and with its social media followers. Online surveys have the advantage of respondents being able to complete it at a time convenient to them and can be time effective for researchers. However, study respondents are not necessarily representative of wider populations, as was the case in this study as we focused on those already connected with or interested in nature. However, the results are not dissimilar from those mentioned above from the RSPB survey [36], the People and Nature Survey [8] and the Ramblers survey [37]. We would expect this sample of people to continue to engage with nature during the pandemic where possible and gain benefits from this engagement. The numbers completing the survey were considerable and this may be due to people noticing nature more during the Covid-19 restrictions, having fewer things to do or wanting to highlight how their connection with nature had changed positively or negatively during the first part of the pandemic. Forestry England spent time trying to encourage people to enjoy and connect with nature during the Covid-19 restrictions. Environment sector organizations could do more to publicise the use of nature for physical activity, connection to nature and the importance for wellbeing and target this at those who are less active, outlining small steps that can be taken and built on. The sample in this study was not diverse in terms of ethnicity or from those in the more deprived areas of England. We did find that those who were less active were more likely to report across all the barriers outlined in the survey. The key barrier of overcrowding is also an issue as access to nature is unevenly distributed across the country with those in urban and peri-urban areas finding it more difficult to remain socially distanced when they cannot travel to other areas as outlined by Shoari et al. [9]. Therefore, addressing issues of inequality of access to good quality nature is increasingly important. There is a very strong focus on woodland creation and expansion in England at present [46] which provides important opportunities for addressing some of the inequalities in access to nature if expansion and creation of woodlands can take place close to, and within easy reach of, large diverse populations in order to provide a range of societal benefits. Supportive programmes should be created to focus on how to meet the demand for access to nature for both wellbeing and nature connection. The results from this research highlight the policy importance of nature near to where people live, that is easily accessible. Strategies to encourage physical activity should make use of the natural environment as a motivation for people to be active highlighting the wide range of benefits that can be gained.

## 5. Conclusions

In this paper we outlined the results from a survey exploring physical activity, engagement and connection with nature, and wellbeing during the Covid-19 restrictions for those who already had an interest in the natural environment. Our research highlights that nature provides critical opportunities and infrastructure to be physically active and at the same time people can gain a wide range of benefits from mental wellbeing, physical wellbeing and a feeling of escape and freedom [47,48]. These benefits need to be taken note of more than ever and should be translated into practical policies that encourage access and benefits for all. Disasters such as the Covid-19 pandemic reveal and make more stark inequalities in a number of key areas concerning health, work and leisure. As with other research in this area, it is crucial in the recovery period from Covid-19 to explore some fundamental questions across society and one of these concerns who gets to access good quality nature near to where they live. There are calls for a fairer and greener society [49] and a changing relationship with nature that is appreciated, valued and given more prominence in the role that it can play in societal wellbeing.

## Figures and Tables

**Table 1:** Demographic data for respondents.

Demographic Category	Number
Male	531
Female	1562
Other	2
**Age**	**Age**
16–24	19
25–34	234
35–44	495
45–54	476
55–64	413
65+	244
Urban	1245
Peri-urban	328
Rural	443
**Ethnicity**	**Ethnicity**
White	2068
Asian or Asian British	15
Black or Black British	5
Mixed/multiple ethnic group	17
Other ethnic group	10
**Index of Multiple Deprivation**	**IMD**
1	65
2	74
3	135
4	156
5	234
6	254
7	256
8	291
9	278
10	274

Totals within demographic category differ depending on completion response (total *n*—2115 individuals. IMD 1 category is the most deprived while category 10 is the least deprived).

## Data Availability

Data is not made available at present due to the on-going nature of the study.

## Supplementary Materials

The following are available online at https://www.mdpi.com/article/10.3390/ijerph18094585/s1, Survey questions used in the study.
