# Supplementary material for: Physical Activity Supporting Connection to Nature, and Helping to Maintain Wellbeing during the Covid-19 Restrictions in England
Source: Int J Environ Res Public Health. 2021 Apr 26;18(9):4585. doi: 10.3390/ijerph18094585 (PMC8123673; doi:10.3390/ijerph18094585)
Supplement: Supplementary file 1 [file ijerph-18-04585-s001.zip › ijerph-1149406-supplementary.pdf]

Supplementary Material

This appendix provides the questions asked in the survey.

1) Which of the following type(s) of green and natural spaces have you visited before and since the Coronavirus (Covid-19) restrictions came into place? Please select all types of places visited for each column

	**Visits Made in the Twelve Months before Covid-19 Restrictions Began**	**Visits since the Covid-19 Restrictions Came into Place on 23 March 2020**
Urban green space (such as a park, field or playground)		
Grounds of a historic property or country park		
Allotment or community garden		
Woodland or forest		
River, lake or canal		
Hill, mountain or moorland		
Beach/other coastline/sea		
Nature/wildlife reserve		
Fields, farmland, countryside		
Another green and natural space		
None of the above		

2) Has the amount of time you have spent on any of the following changed due to Covid-19 restrictions? (answer on a scale from significantly increased to significantly decreased or not applicable)

- Exercising outdoors
- Viewing nature /wildlife
- Viewing trees specifically e.g. in your street, garden, local park, woods, forests
- Visiting a local wood, forest
- Being in your garden
- Tending your window box plants / indoor plants
- Visiting a nature area (not a woodland/forest)

3) How likely are you to continue with any of the above changes in the long term? (answer on a scale from yes definitely to no definitely not or not applicable)

4) To what extent has the following changed or not due to the Covid-19 restrictions? (answer on a scale from significantly increased to significantly decreased or not applicable)

- Your feeling of connection to nature
- Your level of happiness when in nature
- The amount of time you take to appreciate nature

5) On average how often have you visited woodlands or forests for any reason? Select one option in each column only.

	**Visits Made in the Twelve Months before Covid-19 Restrictions Began**	**Visits since the Covid-19 Restrictions Came into Place on 23 March 2020**
Everyday		
4–6 times a week		
1–3 times a week		
1–3 times a month		
4–6 times a year		
1–3 times a year		
Less than 1–2 times per year		
Not applicable		

6) Have you accessed a Forestry England public forest?

	**I Have Accessed a Forestry England Public Forest in the 12 Months before the Covid-19 Restrictions Began**	**I Have Access a Forestry England Public Forest since the Covid-19 Restrictions Began**
Yes		

No		
Not sure		

7) Has your appreciation of the following changed since the Coronavirus restrictions came into place? (options: significant increase, some increase, stayed the same, some decrease, significant decrease, not applicable)

- Trees in my garden
- Trees in my, and nearby, streets
- Trees in my local park
- Trees along footpaths/waterways
- Woodlands/forests

8) Have any of the following motivations for you visiting nature changed because of the Covid-19 restrictions? (options: significant increase, some increase, stayed the same, some decrease, significant decrease, this is not a motivation for me, not applicable)

- For mental health and wellbeing
- To get fresh air
- Wanting to do something as I was not working
- To make the most of the one hour allowed for exercise (e.g. in the first phase of Covid-19 restrictions)
- To walk a dog
- To connect to nature/wildlife
- To take children outside
- To get a break from TV/other electronic devices
- To learn something new/challenge myself
- There was a bit more time as schools were closed
- To take a lunch break/break from work
- To enjoy the spring weather
- To explore new local nature spaces
- For physical health and exercise

9) Have any of the following changed from your engagement with nature since the Covid-19 restrictions? (options: significant change for the better, some change for the better, stayed the same, some change for the worse, significant change for the worse, not applicable)

- Mental wellbeing benefits
- A sense of fun and enjoyment
- I learnt something new/challenged myself
- Gaining a sense of solace from engaging with nature
- Enjoyed my activity with friends
- Sensory stimulation (e.g. sight, sound, smells)
- Enjoyed my activity with family in my household
- Feeling close to nature
- A feeling of escape and freedom
- Physical wellbeing

10) Thinking about the time since Covid-19 restrictions were introduced have any of the following reasons prevented you spending time outside in green and natural spaces? (option: these reasons prevent me spending time outside—pick all that apply)

- I am not leaving home at all
- I am concerned about over crowding and not being able to keep my distance from people
- My nearest accessible green space is too far away
- I do not have the time to get out to green and natural spaces
- I cannot meet people I would usually meet
- I would not be able to use the facilities I need such as public toilets or benches
- I have difficulty getting to accessible green spaces
- Current coronavirus restrictions
- I am worried about breaking the coronavirus restrictions
- I think access to local green and natural places has been restricted

11) What is the total amount of moderate to vigorous physical activity that you have undertaken in the last seven days?

- 150 min or over (2.5 h)
- 60–149 min
- 30–59 min

- Less than 30 min

12) In the past 7 days have you done a continous walk lasting at least 10 min?
    - Yes
    - No
13) In the past 7 days on how many days did you do a walk of at least 10 min? (options: 1 day through to 7 days)
14) How much time did you usually send walking on each day that you did that activity
    - Hours per day
    - Minutes per day
15) Was the effort you put into walking usually enough to raise your breathing rate?
    - Yes
    - No
16) In the past 7 days have you done cycle ride lasting at least 10 min?
    - Yes
    - No
17) In the past 7 days on how many days did you do a cycle ride of at least 10 min? (options: 1 day through to 7 days)
18) How much time did you usually send cycling on each day that you did that activity
    - Hours per day
    - Minutes per day
19) Was the effort you put into cycling usually enough to raise your breathing rate?
    - Yes
    - No
20) In the past 7 days have you done sport, fitness activity (other than walking or cycling) or dance (this would include a virtual class for example)?
    - Yes
    - No
21) In the past 7 days on how many days did you do sport, fitness or dance? (options: 1 day through to 7 days)
22) How much time did you usually send doing sport, fitness activities or dance on each day that you did that activity
    - Hours per day
    - Minutes per day
23) Was the effort you put into sport, fitness or dance usually enough to raise your breathing rate?
    - Yes
    - No
24) What sport, fitness or dance activity have you done in the past 7 days?
    - Gym, exercise machines, weight training, body weight exercises such as press ups, squats, core training etc. (this includes doing these activities at home)
    - Home activity, fitness or exercise class not viewed online or on TV, DVD or video (includes yoga, pilates, circuits)
    - Racket sports (such as tennis, badminton, squash etc.)
    - Water sports (such as surfing, rowing, sailing etc.)
    - Outdoor activities (such as fishing, climbing, mountaineering, orienteering etc.)
    - Swimming
    - Combat, martial arts or any target sports (such as shooting, archery etc.)
    - Dance (this includes online, TV, DVD or video led dance activity)
    - Golf
    - Team sports (such as football, cricket, rugby etc.)
    - Informal physical activity (an activity that was undertaken because it was fun or social e.g. playing running games with your children.)
    - Gymnastics- (including trampolining or cheerleading)
    - Home activity, fitness or exercise class viewed online or on TV, DVD or video (includes yoga, pilates, circuits etc.)
    - Running or jogging
    - Other (please specify)

25) Has the amount of physical activity you have been doing changed or not since the restrictions were introduced? Are you doing: (options: much more than usual, a bit more than usual, about the same, a bit less than usual, much less than usual, not applicable). Please tell us about any of your experiences of green and natural spaces (including trees and woodlands) that have change or are different due to Covid-19 restrictions? (Open question)
26) Overall how satisfied are you with your life nowadays? (option: on a scale from 0 not at all satisfied to 10 completely satisfied)
27) Overall how worthwhile are the things you do in your life? (option: on a scale of 0 not worthwhile to 10 completely worthwhile)
28) Overall how happy did you feel yesterday? (option: on a scale from 0 not at all happy to 10 completely happy)
29) Overall how anxious did you feel yesterday? (option: on a scale from 0 not at all anxious to 10 completely anxious)
30) How happy were you feeling before the Covid-19 restrictions began? (option: on a scale from 0 not at all happy to 10 completely happy)
31) How anxious were you feeling before the Covid-19 restrictions began? (option: on a scale from 0 not at all anxious to 10 completely anxious)
32) Are you? Female, Male, Non-binary
33) Which of the following options best describes your employment status at the present time?
    - Employed
    - Employed but currently furloughed
    - Self-employed
    - Unemployed
    - Looking after home or family
    - In education school/college/university
    - Retired
    - Volunteering
    - Other
34) Which of these ethnic groups do you consider you belong to?
    - White
    - Mixed/multiple ethnic group
    - Black or Black British
    - Asian or Asian British
    - Other ethnic group
35) How many people are there in your household excluding you? (if none state zero)
36) How many children in your household below 16 years of age (if none please state zero)
